# Prevalence and predictors of poor self-care behaviors in patients with chronic heart failure

**DOI:** 10.1038/s41598-024-52611-5

**Published:** 2024-01-23

**Authors:** Yirga Legesse Niriayo, Bisrat Yemane, Solomon Weldegebreal Asgedom, Gebrehiwot Teklay, Kidu Gidey

**Affiliations:** Department of Clinical Pharmacy, School of Pharmacy, College of Health Sciences, Mekelle University, Mekelle, Tigray Ethiopia

**Keywords:** Cardiology, Health care

## Abstract

Despite the indispensable role of self-care behavior in managing heart failure, the practice of self-care behavior remains poor, especially in developing countries. There is a scarcity of research focusing on poor self-care behavior and its determinants within our specific context. Therefore, the objective of this study was to investigate the prevalence and predictors of poor self-care behavior among ambulatory heart failure patients. A facility-based cross-sectional study was conducted at a tertiary care hospital in Ethiopia, involving patients with heart failure. We utilized the European Heart Failure Self-Care Behavior Scale (EHFScBS-9) to evaluate adherence to self-care behaviors. Data were gathered through patient interviews and a review of medical records. A binary logistic regression analysis was performed to identify predictors of poor self-care behavior in heart failure patients. We included a total of 343 participants in the final analysis of this study. The findings revealed that a majority of the patients (73.8%) demonstrated poor overall self-care behavior. Specifically, the majority of patients did not engage in regular exercise (76.1%), failed to consult doctors in case of rapid weight gain (75.6%), did not monitor weight daily (71.5%), did not restrict fluid intake (69.9%), and did not contact doctors in case of experiencing fatigue (68.6%). Additionally, 32.4% of patients did not reach out to doctors when experiencing shortness of breath, 30% did not restrict salt intake, 29% did not adhere to prescribed medication, and only 7% did not consult doctors if edema occurred. Our findings indicated that rural residence (AOR: 5.76, 95% CI: 2.47–13.43), illiteracy (AOR: 2.64, 95% CI: 1.52–6.31), prior hospitalization (AOR: 2.09, 95% CI: 1.21–3.61), and taking five or more medications (AOR: 1.83, 1.01–3.33) were significant predictors of poor self-care behavior. In conclusion, a majority of the participants in our study demonstrated poor self-care behavior. Risk factors for this behavior included rural residence, illiteracy, prior hospitalization, and taking five or more medications. Therefore, it is crucial to prioritize these high-risk patients and implement interventional programs aimed at improving self-care behaviors and overall treatment outcomes in heart failure patients.

## Introduction

Heart failure (HF) is a complex and life-threatening clinical syndrome that results in significant morbidity and mortality, impaired functional capacity, diminished quality of life, and substantial financial burden^1^. It poses a growing global challenge, affecting more than 64 million individuals worldwide^2,3^. The prevalence of HF and its associated health impact has been steadily increasing, particularly among the elderly population and in developing nations^2^. The consequences of HF are severe, encompassing a staggering loss of 9.91 million disability-adjusted life years globally, accompanied by a significant financial expenditure of 346.17 billion US dollars^2^. Although the prognosis of HF has improved over time, mortality rates remain elevated, especially in developing countries^4,5^. Across various continents, the overall mortality of HF is reported to be 16.5%, with the highest rates observed in Africa (34%)^4^. This alarming trend is also evident in Ethiopia, where HF has become a major concern, contributing to a significant in-hospital mortality rate ranging from 17.2 to 24.4%^4,6,7^. The challenges in addressing this condition in Ethiopia include limited access to healthcare resources, inadequate awareness, and a lack of early diagnosis and management options^8^.

Management of HF consists of both non-pharmacological and pharmacologic therapies^9,10^. Adherence to both pharmacologic and non-pharmacologic recommendations is vital to the welfare of HF patients^11^. Self-care behavior (SCB) is an important component of HF management, and it refers to an activity undertaken by patients to care for themselves to promote health and well-being^12,13^. SCB in patients with HF includes adherence to medication, salt-restriction, weight monitoring, fluid restriction, physical activity, and behaviors such as seeking assistance when symptoms occur^13^. SCBs are crucial to reduce readmission rates, complications, and mortalities in HF patients^14,15^. Several randomized clinical trials revealed that SCB reduces HF-related readmissions by 40–50%, and the combined end point of morbidity and mortality by 35%^15,16^.

Despite the significant advances in both pharmacologic and non-pharmacologic therapies, HF remains the leading cause of mortality, morbidity, and healthcare expenditure burden^3^. Lack of adherence to SCBs is one of the most important contributing factors for frequent hospital admissions, mortalities, and healthcare expenditures associated with HF^3,17^. The practice of SCB remains poor, especially in developing countries such as Ethiopia^18–21^. Previous studies reported that the rate of adherence to SCBs ranged from 12 to 40%^20–23^. Adherence to SCB can be influenced by many factors, including gender, age, education, knowledge about disease and treatment, health literacy, access to health care, income, New York Heart Association class (NYHA), comorbidity, and number of medications^24–27^.

In our specific setting, little is known regarding the prevalence of poor SCBs and their contributing factors. To address this knowledge gap, it is imperative to conduct a study that can identify, quantify, and document these issues in patients with HF. Such a study would not only offer valuable insights into the scope of the problem but also highlight existing gaps in healthcare practices. Furthermore, it would increase awareness among healthcare professionals and policymakers, prompting them to prioritize efforts aimed at improving SCBs. Therefore, the main objective of this study was to investigate the prevalence of poor SCBs and identify associated factors among ambulatory HF patients.

## Methodology

### Study design and setting

A facility-based cross-sectional study was conducted between December 2018 and March 2019 at Ayder Comprehensive Specialized Hospital (ACSH), which is the largest public hospital in the Tigray region of Northern Ethiopia. It is a teaching and referral hospital at the College of Health Science, Mekelle University, which provides service for about 10 million people in the catchment area^28,29^.

### Study participants

The source population for this study consisted of patients with HF who received follow-up care at the Ayder Comprehensive Specialized Hospital (ACSH) cardiac clinic. Patients diagnosed with heart failure, aged 18 years or older, and who had a minimum follow-up duration of 6 months with at least one prescribed medication were included in the study. Patients who were too ill to complete the interview, declined to provide consent, or had incomplete medical records were excluded. The participants were randomly selected during their scheduled follow-up appointments.

### Variables and measurements

The study assessed self-care behavior (SCB) as the dependent variable. The independent variables encompassed sociodemographic characteristics (including age, sex, educational level, marital status, residence, and social drug use), as well as clinical and treatment-related factors (such as prior hospitalization, comorbidity, disease severity, treatment duration, and number of medications).

To assess patients’ adherence to self-care behaviors (SCBs), we employed the European Heart Failure Self-Care Behavior Scale (EHFScBS-9) questionnaire, which has been validated in numerous studies^30–32^. This self-reported questionnaire consists of nine items, each rated on a 5-point Likert scale ranging from 1 (‘‘completely agree’’) to 5 (‘‘completely disagree’’). The nine items can be grouped into two dimensions: consulting behaviors and adherence to the regimen. The consulting behaviors dimension explores how frequently individuals with heart failure contact their doctor or nurse when experiencing symptoms such as shortness of breath, swelling (edema), weight gain, and fatigue. On the other hand, the adherence to the recommended regimen dimension evaluates the frequency of self-care activities such as weighing themselves, restricting fluid intake, following a low-salt diet, taking medications regularly, and engaging in exercise. The total possible score on the EHFScBS-9 is 9 to 45, with lower scores indicating better self-care. To facilitate interpretation, the responses were standardized to a possible score range of 0 to 100. In the standardized score of the EHFScBS-9 (0–100), a higher score signifies better self-care. To categorize the level of self-care, patients with an EHFScBS-9 score of ≥ 70 were classified as having good self-care behavior, while those with a score of < 70 were considered to have poor self-care behavior^33^.

### Data collection instrument and procedure

We developed a comprehensive data collection tool consisting of a questionnaire and a data abstraction checklist to gather all necessary information. The questionnaire included socio-demographic factors and SCB, while the data abstraction checklist focused on clinical and treatment-related characteristics. To ensure accuracy and consistency, the questionnaire was translated into the local language (Tigrigna) and then back-translated into English. This process guaranteed the preservation of meaning across translations. Additionally, the Tigrigna-language version of the questionnaire underwent content validity evaluation by a panel of four experts in the field, including two experts from the clinical pharmacy department, one expert from the internal medicine department, and one expert from the public health department. The Tigrigna-language version questionnaire demonstrated good reliability with a Cronbach’s α of 0.86. Before the actual data collection, a pre-test was conducted on 5% of the sample population to assess the face validity of the drafted questionnaire. To carry out the data collection for this study, we employed fifth-year clerkship pharmacy students who received comprehensive training and orientation.

### Bias

To mitigate any potential biases, we implemented various measures during the participant enrollment process. Firstly, we selected participants randomly in order to minimize selection bias. Additionally, in order to address participant bias, we extensively explained the study’s objectives and reassured participants about the confidentiality of their responses. To overcome recall bias, we referred to patients’ medical records for accurate information. Furthermore, a pilot test was conducted to identify and rectify any potential sources of bias or ambiguity in the questionnaire. The questionnaire was evaluated for its content validity by a panel of four experts in the field. As a result, a well-designed and validated questionnaire, incorporating clear and unbiased language, was developed. Finally, interviewers received comprehensive training to ensure consistency in data collection techniques, thus minimizing any potential interviewer bias.

### Study size

The sample size for this study was determined using the single population proportion formula for the prevalence of SCB. Accordingly, a sample of 385 subjects was calculated, assuming a prevalence of SCB of 39.8% based on a previous study^23^, a 95% confidence level, a 5% margin of error, and a 5% contingency for nonresponse rate. Out of the 385 participants approached, 343 patients were included in the final analysis of the study. A simple random sampling technique was employed to recruit subjects for the study.

### Data analysis

The data were checked, cleared, and entered into EPI data management (version 4.2.0). Subsequently, the data were exported to the Statistical Package for Social Science (SPSS version 24.0) for analysis. Descriptive statistics were used to determine the frequency of categorical variables and the mean (standard deviation) of continuous variables. A logistic regression model was used assess factors associated with poor SCB. We checked the model fitness using the Hosmer–Lemeshow test. Multicollinearity was assessed to test the correlation among predictor variables using the variance inflation factor (VIF), and no collinearity was found. The association of each independent variable with poor SCB was determined using bivariate logistic regression analysis. Furthermore, the variables with a p-value < 0.25 in the bivariate analysis were re-entered into a multivariate binary logistic regression model to control for possible effects of the confounders. Finally, the results were reported in terms of an adjusted odds ratio (AOR) with a 95% confidence interval (CI), and a p-value of < 0.05 was considered statistically significant in all analyses.

### Ethics

Our study obtained ethical approval from the Ethics Review Committee (ERC1227/2018) of the School of Pharmacy, College of Health Sciences, Mekelle University. We duly informed all study participants about the study’s objectives and protocol, and written informed consent was obtained from each participant. The confidentiality of personal information was rigorously maintained throughout the study. All procedures were conducted in accordance with the approved institutional guideline.

### Patient and public involvement

Patients or members of the public were not directly involved in the design or planning of this study as the study focused on the HF-SCB.

## Result

### Socio-demographic characteristics

Among the 385 participants approached, 42 patients were excluded from the study due to critical illness preventing them from giving a response^11^, unwillingness to give consent^18^, and incomplete medical records^13^. Therefore, a total of 343 patients were included in this study. The participants had a mean age of 54.16 (SD = 16.42), and more than half (56.3%) were males. The majority of the participants were urban residents (68.5%) and married (74.6%), while 41% were illiterate. In terms of social drug use, 19.8% were alcohol consumers, and 6.7% were cigarette smokers. The findings also showed that 20.4% of the participants used traditional medicine for their illnesses (Table 1).

**Table 1:** Sociodemographic characteristics of patients with heart failure (n = 343).

Variables	Number (%)
Sex	
Male	193 (56.3)
Female	150 (43.7)
Age	
18–35	43 (12.5)
35–60	166 (48.4)
> 60	134 (39.1)
Residence	
Rural	108 (31.5)
Urban	235 (68.5)
Educational level	
Illiterate	141 (41.1)
Primary education	82 (23.9)
Secondary education	65 (19)
College and above	55 (16)
Marital status	
Single	47 (13.7)
Married	256 (74.6)
Divorced	17 (5)
Widowed	23 (6.7)
Traditional medication use	
No	273 (79.6)
Yes	70 (20.4)
Alcohol consumption	
No	275 (80.2)
Yes	68 (19.8)
Cigarette smoking	
No	320 (93.3)
Yes	23 (6.7)

### Clinical characteristics

Among the participants, nearly two-thirds (60.1%) had been hospitalized once or more in the past year, and 74.1% had one or more comorbidities. Around 44.3% of the participants had been receiving treatment for more than two years, and 34.1% were taking five or more medications. The majority of the participants fell under NYHA class II (46.4%) or NYHA class III (43.1%). The most commonly identified comorbidities in patients with HF were valvular heart diseases (21.3%), ischemic heart diseases (20.4%), diabetes (19.2%), and hypertension (17.2%) (Table 2).

**Table 2:** Clinical and treatment related characteristics of patients with heart failure (n = 343).

Variable	Number (%)
prior hospitalization within 1 year	
No	137 (39.9)
Yes	206 (60.1)
Duration on treatment	
< = 2 year	191 (55.7)
> 2 year	152 (44.3)
NYHA class	
I	25 (7.3)
II	159 (46.4)
III	148 (43.1)
IV	11 (3.2)
Co morbidity	
No	89 (25.9)
Yes	254 (74.1)
Number of medication	
< 5 year	26 (65.9)
> = 5 year	117 (34.1)
Left ventricular ejection fraction	
< = 40%	244 (71.1)
> 40%	99 (28.9)
Major comorbidities	
Valvular heart disease	73 (21.3)
Ischemic heart disease	70 (20.4)
Diabetes	66 (19.2)
Hypertension	59 (17.2)
Atrial fibrillation	27 (7.9)

### Prevalence of poor self-care behavior in heart failure patients

Our findings revealed that a majority of the patients (73.8%) exhibited poor overall self-care behavior. With regard to the specific SCBs, the majority of patients did not engage in regular exercise (76.1%), did not consult doctors in case of rapid weight gain (75.6%), failed to monitor weight daily (71.5%), did not restrict fluid intake (69.9%), and did not contact doctors in case of experiencing fatigue (68.6%). Moreover, a considerable percentage of patients (32.4%) did not reach out to doctors when experiencing shortness of breath, 30% did not limit salt intake, and 29% did not adhere to prescribed medication. Only 7% failed to consult doctors if edema occurred (Table 3).

**Table 3:** Self-care behavior among patients with heart failure (n = 343).

Tools	Completely agree, n (%)	Agree, n (%)	Uncertain, n (%)	Disagree, n (%)	Completely disagree, (n%)
I weigh myself every day	30 (8.7)	68 (19.8)	174 (50.7)	68 (19.8)	3 (0.9)
If I gain weight more than 2 kg in one week I contact my doctor	7 (2)	77 (22.4)	176 (51.3)	82 (23.9)	1 (0.3)
If SOB occur I contact my doctor	30 (8.7)	202 (58.9)	99 (28.9)	12 (3.5)	0
If edema occurs I contact my doctor	101 (29.4)	219 (63.8)	23 (6.7)	0	0
I limit the amount of fluids	3 (0.9)	100 (29.2)	187 (54.5)	53 (15.5)	0
If I experienced fatigue I contact my doctor	2 (0.6)	101 (29.4)	192 (56)	48 (14)	
I eat a low-salt diet	73 (21.3)	167 (48.7)	95 (27.7)	8 (2.3)	0
I take my medication as prescribed	118 (34.4)	80 (36.6)	48 (14)	4 (1.2)	0
I exercise regularly	6 (1.7)	76 (22.2)	120 (35)	139 (40.5)	2 (0.6)
Over all self-care behavior					
Good self-care behavior, n (%)			90 (26.2)		
Poor self-care behavior, n (%)			253 (73.8)		

### Factors associated with self-care behavior

A bivariate logistic regression analysis was conducted to compare patients with good SCB and poor SCB based on socio-demographic, clinical, and treatment-related characteristics. Accordingly, rural residence (COR: 7.88, 95% CI: 3.5–17.73), traditional medicine use (COR: 2.49 95%CI: 1.21–5.10), illiteracy (COR: 3.09, 95%CI: 1.21–5.75), prior hospitalization (COR: 2.24, 95%CI: 1.37–3.65), and having five or more medications (COR: 1.85, 1.08–3.20) were significantly associated with poor SCB.

Furthermore, variables with a p-value less than 0.25 in the bivariate analysis underwent further analysis in the multivariate logistic regression model. The overall model, consisting of all predictors, was statistically significant (Chi-square = 62.350, df = 12, p < 0.001). In the multivariate analysis, rural residence (AOR: 5.76, 95% CI: 2.47–13.43), illiteracy (AOR: 2.64, 95% CI: 1.52–6.31), prior hospitalization within one year (AOR: 2.09, 95% CI: 1.21–3.61), and having five or more medications (AOR: 1.83, 95% CI: 1.01–3.33) were identified as independent predictors of poor SCB (Table 4).

**Table 4:** Factors associated with self-care behavior among patients with heart failure (n = 343).

Characteristics	Poor self-care behavior		Bivariate analysis		Multivariate analysis	
	No, (n %)	Yes, (n %)	COR (95% CI)	p-value	AOR (95%CI)	p-value
Sex						
Male	45 (23.3)	148 (76.7)	1	1	1	1
Female	45 (30)	105 (70)	0.71 (0.44–1.15)	0.164	0.68 (0.39–1.18)	0.175
Residence						
Urban	83 (35.3)	152 (64.7)	1	1	1	1
Rural	7 (6.5)	101 (93.5)	7.88 (3.5–17.73)	< 0.001	5.76 (2.47–13.43)	< 0.001
Educational level						
Illiterate	22 (15.6)	119 (84.4)	3.09 (1.52–6.31)	0.002	2.64 (1.21–5.75)	0.014
Primary education	28 (34.1)	54 (65.9)	1.10 (0.54–2.25)	0.790	1.11 (0.51–2.45)	0.788
Secondary education	20 (30.8)	45 (69.2)	1.29 (0.60–2.75)	0.518	1.72 (0.75–3.97)	0.203
College and above	20 (36.4)	35 (63.6)	1	1	1	1
Traditional medication use						
No	80 (29.3.)	193 (70.7)	1	1	1	1
Yes	10 (14.3)	60 (85.7)	2.49 (1.21–5.10)	0.013	1.34 (0.59–3.05)	0.486
Alcohol consumption						
No	76 (27.6)	199 (72.4)	1	1	1	1
Yes	14 (20.6)	54 (79.4)	1.47 (0.77–2.81)	0.239	1.06 (0.50–2.21)	0.885
Hospitalization during the last 1 year						
No	49 (35.8)	88 (64.2)	1	1	1	1
Yes	41 (19.9)	165 (80.1)	2.24 (1.37–3.65)	0.001	2.09 (1.21–3.61)	0.008
NYHA classification						
Class I	10 (40)	15 (60)	1	1	1	1
Class II	36 (22.6)	123 (77.4)	2.28 (0.94–5.50	0.067	1.80 (0.66–4.90)	0.250
Class III	42 (28.4)	106 (71.6)	1.68 (0.70–4.04)	0.245	1.27 (0.46–3.49)	0.640
Class IV	2 (18.2)	9 (81.8)	3 (0.53–16.90)	0.213	1.88 (0.28–12.60)	0.515
Number of medication						
< 5	68 (30.1)	158 (69.9)	1	1	1	1
≥ 5	22 (18.8)	95 (81.2)	1.85 (1.08–3.20)	0.026	1.83 (1.01–3.33)	0.047

## Discussion

Self-care behaviors are essential components of HF management that not only improve the quality of life for HF patients but also reduce morbidity, mortality, and healthcare costs^15^. However, there is limited knowledge regarding the actual adoption of self-care behaviors among HF patients. To address this knowledge gap, it is proposed that assessing an individual's HF-specific self-care behavior (HF-SCB) and its determinants can provide valuable insights to healthcare professionals and practitioners involved in HF management. By evaluating SCBs and the factors that influence them, clinicians can gather crucial information about patients’ adherence to SCBs and identify areas that require intervention or improvement.

Despite the proven morbidity and mortality benefits of SCB in HF patients^11,15^, a majority of the patients (73.8%) in our study demonstrated poor overall SCB. This finding is consistent with findings reported from studies conducted in Sudan (72%), Poland (72.2%), and Ethiopia (77.7%)^21,34,35^. Conversely, a survey conducted with black Africans reported a higher prevalence of poor SCB at 88.4%^22^. In contrast, our finding was lower compared to previous studies done in Southeast Ethiopia (46.4%)^36^ and Vietnam (50.9%)^37^. This discrepancy may be attributed to variations in socio-demographics, disease knowledge, healthcare accessibility, and the method used to assess SCB. Thus, implementing intervention programs that include educational initiatives is crucial to enhance HF self-care practices, leading to improved overall HF outcome^13,38,39^.

Engagement in regular physical activity is a class I recommended non-pharmacologic therapy for all stable HF patients^40^. More importantly, there is substantial evidence indicating that regular physical activity is beneficial for stable HF patients^41,42^. Despite the importance of exercise as a crucial component of HF therapy, our study revealed that 76.1% of patients were not regularly engaged in exercise. This finding aligns with similar findings from previous studies conducted in Ethiopia (80.6%) and Sudan (78.7%)^21,34^. The lack of national coordination in promoting physical exercise, absence of physical exercise guidelines, and prevailing misconceptions about exercise among the African population, including Ethiopia, may contribute to this issue^43–45^.

Non-compliance with SCBs, such as daily weight measurement, is associated with unfavorable clinical outcomes, including increased morbidity and mortality^11,46^. Regular weight monitoring is a recommended aspect of HF-SCB, as it enables patients to track their volume status, detect deterioration, and prevent exacerbations^10,47^. However, our study found that 71.5% of patients did not regularly monitor their weight. Many other studies have also reported similar findings^48–50^. Additionally, it is crucial for patients to promptly seek medical assistance if they experience sudden weight gain exceeding 2 kg within a week^51^. Surprisingly, our study discovered that a significant majority (75.6%) of participants failed to reach out to their doctors in such instances of rapid weight gain. This may be attributed to the fact that most patients do not perceive weight gain as a symptom of deteriorating HF and therefore do not associate it with adverse outcomes related to HF^52^. Consequently, implementing intervention programs that emphasize education could prove beneficial in encouraging patients to engage in HF-SCB, including regular weight monitoring, ultimately improving overall HF prognosis^46^.

Fluid overload is associated with adverse outcomes in patients with HF and is responsible for a substantial number of hospital admissions^10,53^. Therefore, patients with HF are often advised to limit their fluid intake to a maximum of 1500 ml/day^54,55^. However, this study found that more than two-thirds (69.9%) of the patients did not restrict their fluid intake, which is consistent with a previous study conducted in Ethiopia^56^. The reason for this might be the misconception among HF patients regarding fluid intake, as revealed by a previous study where more than one-third of the patients believed that they should drink plenty of fluids as part of HF-SCB^52^.

Among the HF-SCBs, medication adherence is the most important activity for effective management of HF^57,58^. However, it remains challenging in HF patients^59,60^. Our study revealed that 29% of the participants were non-compliant with their prescribed medication. This finding aligns with similar results reported in a South African study (29%)^20^ and a study conducted in Sudan (25%)^34^. For patients with HF, salt restriction is commonly recommended to prevent fluid retention, symptoms exacerbation, and HF decompensation^10,40^. In our study, approximately 30% of the participants did not adhere to the low-salt diet recommendation, aligning with findings from other similar studies^21,61,62^. On contrary, our finding was lower compared to finding reported in Sudan (72.3%)^34^.

Moreover, early recognition of symptoms and prompt response to worsening symptoms are important aspects of HF self-care behaviors, as they can improve the patients' quality of life and prevent unnecessary hospitalizations^63,64^. In HF patients, the inability to respond to symptoms and delayed seeking of treatment for worsening symptoms contribute to unnecessary hospitalizations, poor quality of life, and increased morbidity and mortality^63,65^. Several studies reported that HF patients delay for several days before seeking care for HF symptoms^64–66^. In our study, while the majority of patients contacted their doctors if they experienced edema and shortness of breath, 68.6% failed to contact their doctors for fatigue. This lack of response might be attributed to insufficient symptom monitoring, waiting to see if the symptoms would improve, or perceiving the symptom as minor^65,66^ Therefore, it is crucial to educate HF patients about recognizing these warning symptoms to enable them to take appropriate and timely action^40^.

Several studies have demonstrated that non-adherence SCB is associated with a high risk of hospitalization in HF patients^14,67–69^. Similarly, in our study, prior hospitalization was significantly associated with poor SCB. Patients who have been hospitalized one or more times in the last year were two times more likely to have poor SCB than those who have not been hospitalized. Moreover, patients who took five or more medications were more likely to have poor SCB compared to those who took a lower number of medications. This could be explained by the fact that patients with multiple medications may be reluctant to take their medications appropriately due to pill burden, safety, and cost issues^70^. Additionally, the level of education has been found to be significantly associated with SCB in different studies^19,24,26,71,72^. Similarly, participants who could not read and write were 2.6 times more likely to have poor SCB compared to those who attended college and above in our study. This could be justified that participants with better education have better capability of accessing information related to SCB while illiterate patients may need more time to learn self-care^71^.

Our study also found that rural residents were approximately six times more likely to have poor SCB compared to their urban counterparts. This finding is consistent with a similar study conducted in Iran^73^. The reasons for this disparity can be attributed to various factors, including rural–urban inequalities in socioeconomic resources and access to healthcare^74,75^. Furthermore, inadequate health literacy, limited access to healthcare services, negative perceptions of modern medicine, and a greater reliance on traditional and spiritual healers among rural populations could contribute to this trend^76,77^. Therefore, healthcare providers and policymakers need to be aware of these challenges in the rural population and design specific strategies to meet their needs, helping them develop tailored self-care management plans based on their unique circumstances.

Finally, it is important to acknowledge certain limitations of our study. The cross-sectional nature of our study may not establish causality between non-adherence to SCB and its contributing factors. Patients may understate socially undesirable activities like non-adherence to SCBs due to self-report concerns. Furthermore, the findings of this study should be cautiously extrapolated to other countries, as they depend on factors such as population socioeconomic status, disease distribution, the employed method of SCB assessment, and the healthcare system in place.

## Conclusion

Most of the participants in our study had poor overall self-care behavior. Rural residence, illiteracy, prior hospitalization, and the use of five or more medications were identified as predictors of poor self-care behavior in HF patients. Therefore, more emphasis should be given to these high-risk patients. We recommend implementing further interventional programs to enhance SCBs, and improve overall treatment outcomes in heart failure patients. Moreover, we suggest researchers to conduct longitudinal studies with a more robust design to establish a cause-and-effect relationship between SCB and their determinants.

## Data Availability

The dataset of this study is available from the corresponding author upon request.

## References

[1] Ural D (2015). Diagnosis and management of acute heart failure. Anatol. J. Cardiol..

[2] Lippi, G. & Sanchis-Gomar, F. Global epidemiology and future trends of heart failure. *AME Med. J.* **5**, 10.21037/amj.2020.03.03 (2020).

[3] Savarese G, Lund LH (2017). Global public health burden of heart failure. Cardiac. Fail. Rev..

[4] Tigabe M, Fentahun A, Getawa S, Gelaye KA, Gebreyohannes EA (2021). Clinical characteristics and in-hospital outcome of acute heart failure patients admitted to the medical ward of University of Gondar comprehensive specialized hospital, Northwest Ethiopia. Vasc. Health Risk Manage..

[5] Savarese G (2023). Global burden of heart failure: A comprehensive and updated review of epidemiology. Cardiovasc. Res..

[6] Zeru MA (2018). Assessment of major causes of heart failure and its pharmacologic management among patients at Felege Hiwot referral hospital in Bahir Dar, Ethiopia. J. Public Health Epidemiol..

[7] Tirfe M, Nedi T, Mekonnen D, Berha AB (2020). Treatment outcome and its predictors among patients of acute heart failure at a tertiary care hospital in Ethiopia: A prospective observational study. BMC Cardiovasc. Disord..

[8] Beri B, Fanta K, Bekele F, Bedada W (2023). Management, clinical outcomes, and its predictors among heart failure patients admitted to tertiary care hospitals in Ethiopia: Prospective observational study. BMC Cardiovasc. Disord..

[9] Sánchez LZR, Correa LEE, Figuera FAC (2014). Adherence to phararmacological and non-pharmacological treatment in patients with heart failure/Adherencia al tratamiento farmacológico y no farmacológico en pacientes con falla cardiaca. Enferm. Glob..

[10] Atherton JJ (2016). 2016 ESC guidelines for the diagnosis and treatment of acute and chronic heart failure. Eur. J. Heart Fail..

[11] van der Wal MHL, van Veldhuisen DJ, Veeger NJGM, Rutten FH, Jaarsma T (2010). Compliance with non-pharmacological recommendations and outcome in heart failure patients. Eur. Heart J..

[12] da-Conceição AP, dos-Santos MA, dos-Santos B, da-Cruz DDALM (2015). Self-care in heart failure patients. Rev. Lat. Am. Enfermagem..

[13] Jaarsma T, Abu-Saad HH, Dracup K, Halfens R (2000). Self-care behaviour of patients with heart failure. Scand. J. Caring Sci..

[14] Moser DK (2012). Role of self-care in the patient with heart failure. Curr. Cardiol. Rep..

[15] Ditewig JB, Blok H, Havers J, van Veenendaal H (2010). Effectiveness of self-management interventions on mortality, hospital readmissions, chronic heart failure hospitalization rate and quality of life in patients with chronic heart failure: A systematic review. Patient Educ. Couns..

[16] Jovicic A, Holroyd-Leduc JM, Straus SE (2006). Effects of self-management intervention on health outcomes of patients with heart failure: A systematic review of randomized controlled trials. BMC Cardiovasc. Disord..

[17] von Lueder TG, Agewall S (2018). The burden of heart failure in the general population: A clearer and more concerning picture. J. Thorac. Dis..

[18] Jaarsma T (2013). Comparison of self-care behaviors of heart failure patients in 15 countries worldwide. Patient Educ. Couns..

[19] Macabasco-O'Connell A (2011). Relationship between literacy, knowledge, self-care behaviors, and heart failure-related quality of life among patients with heart failure. J. Gen. Intern. Med..

[20] Ruf V (2010). Medication adherence, self-care behaviour and knowledge on heart failure in urban South Africa: The Heart of Soweto study. Cardiovasc. J. Afr..

[21] Seid MA, Abdela OA, Zeleke EG (2019). Adherence to self-care recommendations and associated factors among adult heart failure patients. From the patients' point of view. PloS one.

[22] N'Cho-Mottoh MP (2015). Assessment of treatment adherence among black Africans with heart failure. Med. Sante Trop..

[23] Beker J, Belachew T, Mekonin A, Hailu E (2014). Predictors of adherence to self-care behaviour among patients with chronic heart failure attending Jimma University Specialized Hospital Chronic Follow up Clinic, South West Ethiopia. J. Cardiovasc. Dis. Diagn..

[24] Sedlar N (2017). Factors related to self-care behaviours in heart failure: A systematic review of European Heart Failure Self-Care Behaviour Scale studies. Eur. J. Cardiovasc. Nurs..

[25] Peters-Klimm F (2013). Determinants of heart failure self-care behaviour in community-based patients: A cross-sectional study. Eur. J. Cardiovasc. Nurs..

[26] Hailu-Gebru T (2020). Self-care behavior and associated factors among heart failure patients in Tigray, Ethiopia: A cross-sectional study. Clin. Nurs. Res..

[27] Zou H, Chen Y, Fang W, Zhang Y, Fan X (2017). Identification of factors associated with self-care behaviors using the COM-B model in patients with chronic heart failure. Eur. J. Cardiovasc. Nurs..

[28] Niriayo YL (2021). Self-medication practice and contributing factors among pregnant women. PLoS One.

[29] Hailu A (2023). Patterns of medical admissions and predictors of mortality in ayder comprehensive specialized hospital, northern Ethiopia: A prospective observational study. Int. J. Gener. Med..

[30] Jaarsma T, Arestedt KF, Mårtensson J, Dracup K, Strömberg A (2009). The European Heart Failure Self-care Behaviour scale revised into a nine-item scale (EHFScB-9): A reliable and valid international instrument. Eur. J. Heart Fail..

[31] Lambrinou E (2014). The Greek version of the 9-item European Heart Failure Self-care Behaviour Scale: A multidimensional or a uni-dimensional scale?. Heart Lung.

[32] Sedlar N (2017). Measuring self-care in patients with heart failure: A review of the psychometric properties of the European Heart Failure Self-Care Behaviour Scale (EHFScBS). Patient Educ. Couns..

[33] Wagenaar KP (2017). Interpretability of the European Heart Failure Self-care Behaviour scale. Patient Prefer. Adher..

[34] Al-khadher MAA, Fadl-Elmula I, Ahmed WAM (2015). Compliance to treatment and quality of life of Sudanese patients with heart failure. Int. J. Prev..

[35] Mlynarska A, Golba KS, Mlynarski R (2018). Capability for self-care of patients with heart failure. Clin. Interv. Aging.

[36] Getachew A, Assefa T, Negash W (2022). Self-care behavior and associated factors among patients with heart failure in public hospitals of Southeast Ethiopia. J. Int. Med. Res..

[37] Huyen, N. N., Jullamate, P. & Kangchai, W. *The First International Conference on Interdisciplinary Research and Development* (2022).

[38] Boren SA, Wakefield BJ, Gunlock TL, Wakefield DS (2009). Heart failure self-management education: a systematic review of the evidence. Int. J. Evid. Based Healthcare.

[39] González B (2014). Educational level and self-care behaviour in patients with heart failure before and after nurse educational intervention. Eur. J. Cardiovasc. Nurs..

[40] Dickstein K (2008). ESC guidelines for the diagnosis and treatment of acute and chronic heart failure 2008: The Task Force for the diagnosis and treatment of acute and chronic heart failure 2008 of the European Society of Cardiology. Developed in collaboration with the Heart Failure Association of the ESC (HFA) and endorsed by the European Society of Intensive Care Medicine (ESICM). Eur. J. Heart Fail..

[41] Rees K, Taylor RR, Singh S, Coats AJ, Ebrahim S (2004). Exercise based rehabilitation for heart failure. Cochrane Database Syst. Rev..

[42] Conraads VM (2012). Adherence of heart failure patients to exercise: Barriers and possible solutions: A position statement of the Study Group on Exercise Training in Heart Failure of the Heart Failure Association of the European Society of Cardiology. Eur. J. Heart Fail..

[43] Klompstra L, Jaarsma T, Strömberg A (2015). Physical activity in patients with heart failure: Barriers and motivations with special focus on sex differences. Patient Prefer. Adher..

[44] Joseph RP, Ainsworth BE, Keller C, Dodgson JE (2015). Barriers to physical activity among african american women: An integrative review of the literature. Women Health.

[45] Kassa MD, Grace J (2018). Barriers to integrate physical exercise into the ethiopian healthcare system to treat non-communicable diseases. Glob. J. Health Sci..

[46] Lu M-X (2016). Weight management belief is the leading influential factor of weight monitoring compliance in congestive heart failure patients. Acta Cardiol. Sin..

[47] Wang X-H (2014). Establishment of a weight management scale for patients with congestive heart failure. Acta Cardiol. Sin..

[48] van der Wal MH, Jaarsma T, Moser DK, van Gilst WH, van Veldhuisen DJ (2007). Unraveling the mechanisms for heart failure patients' beliefs about compliance. Heart Lung.

[49] Jones CD (2014). Self-reported recall and daily diary-recorded measures of weight monitoring adherence: Associations with heart failure-related hospitalization. BMC Cardiovasc. Disord..

[50] Ng'ang’a-Oginga, I. *Heart Failure Knowledge and Self Care Behaviour Practices Among Ambulatory Heart Failure Patients at Kenyatta National Hospital, University of Nairobi* (2016).

[51] Lainscak M (2011). Self-care management of heart failure: practical recommendations from the Patient Care Committee of the Heart Failure Association of the European Society of Cardiology. Eur. J. Heart Fail..

[52] Ni H (1999). Factors influencing knowledge of and adherence to self-care among patients with heart failure. Arch. Internal Med0.

[53] Pellicori P, Kaur K, Clark AL (2015). Fluid management in patients with chronic heart failure. Cardiac Fail. Rev..

[54] Johansson P, van der Wal MH, Strömberg A, Waldréus N, Jaarsma T (2016). Fluid restriction in patients with heart failure: How should we think?. Eu. J. Cardiovasc. Nurs..

[55] Yancy CW (2017). 2017 ACC/AHA/HFSA focused update of the 2013 ACCF/AHA guideline for the management of heart failure: A report of the American College of Cardiology/American Heart Association Task Force on Clinical Practice Guidelines and the Heart Failure Society of America. J. Am. Coll. Cardiol..

[56] Sewagegn N, Fekadu S, Chanie T (2015). Adherence to self-care behaviours and knowledge on treatment among heart failure patients in ethiopia: The case of a tertiary teaching hospital. J. Pharma Care Health Syst..

[57] Ruppar TM, Cooper PS, Mehr DR, Delgado JM, Dunbar-Jacob JM (2016). Medication adherence interventions improve heart failure mortality and readmission rates: Systematic review and meta-analysis of controlled trials. J. Am. Heart Assoc..

[58] Wu J-R, Moser DK (2018). Medication adherence mediates the relationship between heart failure symptoms and cardiac event-free survival in patients with heart failure. J. Cardiovasc. Nurs..

[59] Ho PM, Bryson CL, Rumsfeld JS (2009). Medication adherence: Its importance in cardiovascular outcomes. Circulation.

[60] Wu J-R, Moser DK, Lennie TA, Burkhart PV (2008). Medication adherence in patients who have heart failure: A review of the literature. Nurs. Clin. N. Am..

[61] Nieuwenhuis MM, Jaarsma T, van Veldhuisen DJ, Postmus D, van der Wal MH (2012). Long-term compliance with nonpharmacologic treatment of patients with heart failure. Am. J. Cardiol..

[62] Lennie TA (2008). Relationship of heart failure patients' knowledge, perceived barriers, and attitudes regarding low-sodium diet recommendations to adherence. Progress Cardiovasc. Nurs..

[63] Takei M (2020). Delay in seeking treatment before emergent heart failure readmission and its association with clinical phenotype. J. Intens. Care.

[64] Lam C, Smeltzer SC (2013). Patterns of symptom recognition, interpretation, and response in heart failure patients: An integrative review. J. Cardiovasc. Nurs..

[65] Sethares KA, Sosa M-E, Fisher P, Riegel B (2014). Factors associated with delay in seeking care for acute decompensated heart failure. J. Cardiovasc. Nurs..

[66] Evangelista LS, Dracup K, Doering LV (2000). Treatment-seeking delays in heart failure patients. J. Heart Lung Transplant..

[67] Shojaei F, Ebrahimi SM, Assemi S (2011). Self-care behavior and affecting factors among patients with heart failure in Iran. Saudi Med. J..

[68] Ruppar TM, Cooper PS, Johnson ED, Riegel B (2019). Self-care interventions for adults with heart failure: A systematic review and meta-analysis protocol. J. Adv. Nurs..

[69] Chen IH, Chi MJ (2015). Effects of self-care behaviors on medical utilization of the elderly with chronic diseases—a representative sample study. Arch. Gerontol. Geriatr..

[70] Parsons C (2017). Polypharmacy and inappropriate medication use in patients with dementia: An underresearched problem. Therapeut. Adv. Drug Saf..

[71] Cavalcante LM (2018). Influence of socio-demographic characteristics in the self-care of people with heart failure. Rev. Brasil. Enfermagem.

[72] Santesmases-Masana R, Gonzalez-de Paz L, Hernandez-Martinez-Esparza E, Kostov B, Navarro-Rubio MD (2019). Self-care practices of primary health care patients diagnosed with chronic heart failure: A cross-sectional survey. Int. J. Environ. Res. Public Health.

[73] Bagheri-Saweh MI, Lotfi A, Salawati-Ghasemi S (2018). Self-care behaviors and related factors in chronic heart failure patients. Int. J. Biomed. Public Health.

[74] Lee KS, Moser DK, Pelter MM, Nesbitt T, Dracup K (2017). Self-care in rural residents with heart failure: What we are missing. Eur. J. Cardiovasc. Nurs..

[75] van der Hoeven M, Kruger A, Greeff M (2012). Differences in health care seeking behaviour between rural and urban communities in South Africa. Int. J. Equity Health.

[76] Strasser R, Kam SM, Regalado SM (2016). Rural health care access and policy in developing countries. Annu. Rev. Public Health.

[77] Sav A (2015). Self-management of chronic conditions in a rural and remote context. Austral. J. Primary Health.

